# Acute Outpatient ECT for Depression: Case Series of the First Clinical Pilot in Ireland

**DOI:** 10.1192/bjo.2025.10731

**Published:** 2025-06-20

**Authors:** Saba Inam, Mirat Qari, Henry Roberts, Thomas McMonagle, Martha Finnegan

**Affiliations:** Department of Psychiatry, Tallaght University Hospital, Dublin, Ireland

## Abstract

**Aims:** ECT is a well-evidenced, cost-effective intervention for treatment-resistant depression. In Ireland acute (twice-weekly) outpatient ECT for depression has not been reported, though common elsewhere. Ireland has among the lowest number of inpatient psychiatry beds per person in Europe. We observed a clinical need for acute outpatient ECT for people who could not access elective inpatient care.

**Methods:** All cases provided written informed consent. A multidisciplinary (psychiatry, anaesthesiology, nursing) protocol for assessment and delivery of acute outpatient ECT was developed and implemented, cases described and feedback from stakeholders sought in an acceptability forum.

**Results:** Four medically stable patients (ASA Grades 2) completed acute outpatient ECT, receiving between n=6 and n=14 ECT treatments, attending from home. N=140 inpatient psychiatry bed days were saved, and n=45 community psychiatry reviews were required. No adverse events or medical interventions occurred. Three people had CGI outcome of “very much improved” and one person halted their treatment course when “minimally improved”, citing lack of response. Stakeholder feedback in an acceptability forum highlighted the increased resource intensity of twice weekly community review for outpatient ECT, and the positive outcomes for treatment-resistant depression.

**Conclusion:** Acute outpatient ECT was safe and effective in this case series and resulted in n=140 psychiatry inpatient bed days being saved. There was an increased need for reviews from the community team during the treatment protocol. Medically stable patients with substantial social support were eligible for this pilot phase, thus a priority for future development must be equity of access to this effective intervention.

